# Antibiotic stewardship and safety in office-based transrectal prostate biopsy: a single-center retrospective analysis of complications

**DOI:** 10.25122/jml-2026-0067

**Published:** 2026-04

**Authors:** Osama Salloum, Romica Cergan, Nicoleta Aurelia Sanda, Alex Dick, Costin Petcu, Costin Gingu, Amelia Nicoleta Buturin, Adrian Costache

**Affiliations:** 1 Pathology Department, Faculty of Medicine, Carol Davila University of Medicine and Pharmacy, Bucharest, Romania; 2 Anatomy Department, Faculty of Medicine, Carol Davila University of Medicine and Pharmacy, Bucharest, Romania; 3 University Emergency Hospital Bucharest, Bucharest, Romania; 4 Urology Department, Faculty of Medicine, Carol Davila University of Medicine and Pharmacy, Bucharest, Romania; 5 General Surgery Department, Titu Maiorescu University, Bucharest, Romania

**Keywords:** prostate cancer, MRI–ultrasound fusion biopsy, systematic biopsy, PI-RADS, clinically significant prostate cancer, antibiotic stewardship, radical prostatectomy, surgical pathology

## Abstract

Multiparametric magnetic resonance imaging has improved risk stratification and lesion-directed sampling in suspected prostate cancer. MRI–ultrasound fusion biopsy targets MRI-visible lesions, whereas systematic biopsy provides non-targeted glandular sampling. This study aimed to compare the diagnostic yield of fusion and systematic biopsy in a single-center paired cohort and to explore biopsy-to-surgical pathology concordance in operated patients. This retrospective study included 138 men who underwent both fusion-targeted and systematic biopsy during the same diagnostic work-up. The primary endpoint was overall prostate cancer detection; secondary endpoints included clinically significant prostate cancer detection, discordance between methods, PI-RADS-stratified detection, complications, and exploratory surgical pathology findings. Clinically significant cancer was defined as a Gleason score ≥3+4 / ISUP Grade Group ≥2. Fusion biopsy detected prostate cancer in 65/138 men (47.1%) versus 56/138 (40.6%) for systematic biopsy; combined biopsy detected cancer in 72/138 (52.2%). Fusion-only detection occurred in 16 patients (11.6%) and systematic-only detection in 7 (5.1%). The paired difference for overall cancer detection did not reach statistical significance (exact McNemar *P* = 0.093). Clinically significant prostate cancer was detected in 37/138 men (26.8%) by fusion biopsy and 35/138 (25.4%) by systematic biopsy (*P* = 0.815). Detection rates increased with PI-RADS category and were highest in PI-RADS 5 lesions. Surgical pathology was available in 45 patients; upgrading occurred in 21 (46.7%) and adverse pathology in 18 (40.0%). Fusion biopsy showed a numerically higher overall detection rate, particularly in PI-RADS 5 lesions, while systematic biopsy retained complementary value. These findings support a combined targeted and systematic biopsy strategy in selected patients. Current evidence also suggests that careful antibiotic stewardship is a safe approach in the management of office-based transrectal prostate biopsy.

## INTRODUCTION

Prostate cancer remains one of the most frequently diagnosed malignancies in men and continues to account for substantial cancer-related morbidity [1]. In Romania, cancer mortality trends have been described as an important public health concern, supporting the need for locally generated diagnostic and outcome data [2]. Modern diagnostic pathways increasingly seek not merely to identify prostate cancer, but to preferentially detect clinically significant disease while minimizing the overdiagnosis of indolent tumors [3]. This distinction is critical because treatment decisions are strongly influenced by histological grade, tumor burden, imaging characteristics, and the likelihood that the detected lesion is biologically meaningful.

Systematic prostate biopsy has traditionally served as the standard diagnostic approach in men with suspected disease. Under ultrasound guidance, tissue cores are obtained from predefined areas of the gland according to a non-targeted template. This strategy offers broad sampling and may capture multifocal or MRI-invisible tumors, yet it remains vulnerable to sampling error and may undersample anterior or apical lesions. It may also identify low-volume disease of uncertain immediate clinical relevance [3].

The incorporation of multiparametric MRI into the pre-biopsy pathway has substantially altered prostate cancer diagnostics [4]. MRI not only improves lesion visualization but also provides structured risk stratification through the Prostate Imaging Reporting and Data System (PI-RADS) [5]. MRI–ultrasound fusion biopsy builds upon this information by aligning MRI-defined targets with real-time ultrasonography, thereby enabling lesion-directed sampling [6]. In principle, this technique should increase the yield of clinically meaningful tumors, particularly when the MRI lesion is well characterized and highly suspicious.

Even so, the role of systematic biopsy has not disappeared. Contemporary evidence suggests that targeted and systematic sampling offer complementary information [7]. Targeted biopsy may improve lesion-specific detection, whereas systematic biopsy may still identify tumors that are MRI-invisible, multifocal, or simply missed because of targeting limitations [7,8]. For that reason, the practical question is not only whether fusion biopsy detects more cancer, but also how much incremental value it provides when both methods are applied to the same patient.

Because cancer mortality patterns and diagnostic pathways may vary across health systems, single-center real-world cohorts can provide useful complementary evidence when interpreted within their local epidemiological context [2]. The present study was therefore designed as a single-center paired analysis comparing MRI–ultrasound fusion biopsy with systematic biopsy in the same cohort. The principal aim was to assess the incremental diagnostic yield of fusion biopsy for overall prostate cancer detection. Secondary aims were to compare clinically significant prostate cancer detection, examine concordance and discordance between biopsy methods, evaluate PI-RADS-stratified detection patterns, and explore biopsy-to-surgical pathology concordance in the subgroup of patients who underwent radical prostatectomy.

## MATERIAL AND METHODS

### Study design and setting

This investigation was a retrospective, single-center, paired diagnostic yield study. The paired design was chosen because every included patient underwent both MRI–ultrasound fusion-targeted biopsy and systematic biopsy, allowing a within-patient comparison of the two techniques.

The study period extended from 2023 to 2025. The study was conducted in accordance with the Declaration of Helsinki. Given the retrospective design, informed consent was waived in accordance with institutional policy.

The primary analysis was based on biopsy histopathology, which was available for all included patients. Surgical pathology was not used as a universal reference standard because it was available only for patients who underwent radical prostatectomy. Therefore, analyses involving surgical pathology were considered exploratory rather than confirmatory.

### Patient selection

The final cohort comprised 138 adult men investigated for suspected prostate cancer. Inclusion criteria were pre-biopsy mpMRI, performance of both MRI–ultrasound fusion-targeted biopsy and systematic biopsy during the same diagnostic work-up, and the availability of analyzable histopathological results for both biopsy components.

Patients with incomplete essential biopsy histopathology, duplicate records, or findings that could not be categorized as cancer versus non-cancer were excluded from the analyzable cohort.

### Clinical and imaging variables

The database included demographic, clinical, imaging, biopsy-related, complication-related, and surgical pathology variables. These included age, total prostate-specific antigen (PSA), prostate volume, PSA density, PI-RADS category, digital rectal examination findings where available, fusion biopsy histology, systematic biopsy histology, complication severity, and postoperative pathological variables in operated patients. PSA density was calculated by dividing total PSA by prostate volume and was expressed as ng/mL/cm^3^.

### MRI assessment

All patients underwent pre-biopsy multiparametric MRI according to the institutional prostate MRI protocol using a 3-T scanner. MRI-visible lesions were categorized according to PI-RADS version 2.1 and used to direct fusion-targeted sampling [5]. PI-RADS information was available for 135 of the 138 patients included in the analysis. MRI examinations were interpreted by radiologists experienced in prostate imaging.

### Biopsy procedures

All patients underwent combined MRI–ultrasound fusion-targeted biopsy and systematic biopsy during the same diagnostic episode. Biopsies were performed via the transrectal route, according to institutional practice. In the source database, the non-targeted component was recorded as “random biopsy”; in the manuscript, the term “systematic biopsy” is used because the cores were obtained according to a template-based, non-targeted sampling strategy.

Fusion biopsy was defined as targeted sampling of MRI-suspicious lesions after alignment of the MRI target with real-time ultrasound guidance. Systematic biopsy was defined as non-targeted template sampling of the prostate under ultrasound guidance [3,7]. Patient-level procedural details, including the exact number of targeted and systematic cores, sampling order, anesthesia, and antibiotic prophylaxis, were not consistently captured in the retrospective database.

### Histopathological definitions

Biopsy histopathology was assessed separately for fusion and systematic samples. Adenocarcinoma was classified as cancer. Benign tissue, benign prostatic hyperplasia (BPH), inflammation, high-grade prostatic intraepithelial neoplasia (HGPIN), and atypia/atypical small acinar proliferation (ASAP)-like findings were not considered cancer in the primary dichotomized analysis,although they were preserved descriptively in the source dataset.

Clinically significant prostate cancer was defined a priori as a Gleason score ≥3+4, corresponding to International Society of Urological Pathology (ISUP) Grade Group ≥2 [9].

### Surgical pathology status

A dedicated surgical pathology status variable was introduced to distinguish among patients with available radical prostatectomy pathology, patients without biopsy-detected cancer for whom surgical pathology was not applicable, and patients with biopsy-detected cancer for whom surgical pathology was not available in the database.

This distinction was considered methodologically important because the absence of surgical pathology does not imply the absence of disease. Rather, it reflects either the absence of an indication for radical prostatectomy or the lack of an available postoperative specimen in the study database.

### Outcomes and statistical analysis

The primary endpoint was overall prostate cancer detection by fusion biopsy compared with systematic biopsy. Secondary endpoints included detection of clinically significant prostate cancer, concordance and discordance between methods, PIRADS-stratified detection rates, complications, and exploratory biopsy-to-surgical pathology concordance in patients who underwent radical prostatectomy.

Continuous variables were summarized as medians and interquartile ranges, and categorical variables as counts and percentages. Paired comparisons between fusion and systematic biopsy were performed using the exact McNemar test. Two-sided *P* values <0.05 were considered statistically significant. Data were collected and organized in Microsoft Excel. Statistical analyses were performed using IBM SPSS Statistics version 30. Figures were generated using IBM SPSS Statistics version 30 and Microsoft Excel. Because surgical pathology was unavailable for the full cohort, the principal interpretation focused on diagnostic yield and incremental detection rather than on full diagnostic accuracy.

## RESULTS

### Baseline characteristics

**Figure 1:**
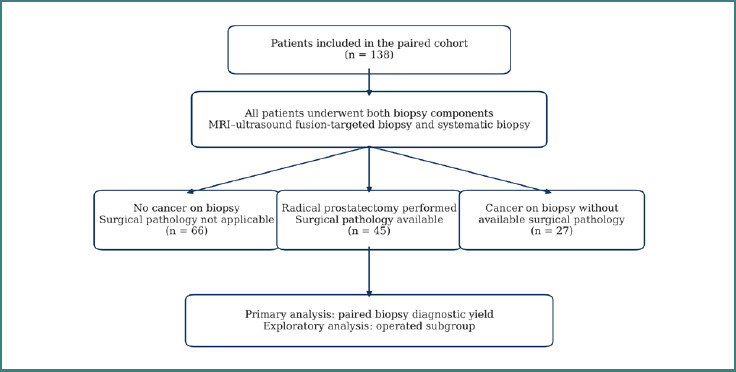
Study flow diagram and classification of surgical pathology status. All included patients underwent both biopsy components, allowing paired comparison of MRI–ultrasound fusion-targeted biopsy and systematic biopsy. Surgical pathology was available only for patients who underwent radical prostatectomy and was therefore interpreted as exploratory rather than as a universal reference standard.

A total of 138 patients were included in the final analysis (Figure 1). Median age was 66 years (interquartile range [IQR], 59–71), median PSA was 7.46 ng/mL (IQR, 5.50–10.00), median prostate volume was 54.5 cm^3^ (IQR, 39.9–76.7), and median PSA density was 0.14 ng/mL/cm^3^ (IQR, 0.085–0.199) (Table 1).

**Table 1:** Baseline characteristics of the study cohort

Variable	Value
Patients, *n*	138
Age, years, median (IQR)	66 (59-71)
PSA, ng/mL, median (IQR)	7.46 (5.50-10.00)
Prostate volume, cm^3^, median (IQR)	54.5 (39.9-76.7)
PSA density, ng/mL/cm^3^, median (IQR)	0.14 (0.085-0.199)
PI-RADS available, *n*	135 (97.8%)
PI-RADS 3, *n* (%)	24 (17.4%)
PI-RADS 4, *n* (%)	80 (58.0%)
PI-RADS 5, *n* (%)	31 (22.5%)
Missing PI-RADS, *n* (%)	3 (2.2%)

Values are presented as median (interquartile range) or *n* (%). Percentages were calculated using the full cohort as the denominator (*n* = 138). IQR, interquartile range; PSA, prostate-specific antigen; PI-RADS, Prostate Imaging Reporting and Data System.

PI-RADS information was available for 135 patients. Of these, 24 patients had PI-RADS 3 lesions, 80 had PI-RADS 4 lesions, and 31 had PI-RADS 5 lesions. Most patients with available PIRADS information, therefore, had PI-RADS 4 or 5 lesions.

### Detection of prostate cancer and clinically significant disease

Fusion biopsy detected prostate cancer in 65 of 138 patients (47.1%), whereas systematic biopsy detected cancer in 56 patients (40.6%). Combined biopsy detected cancer in 72 patients (52.2%). The absolute difference between fusion and systematic biopsy was therefore 6.5 percentage points in favor of fusion biopsy. In paired analysis, this numerical advantage did not reach statistical significance (exact McNemar *P* = 0.093).

**Figure 2:**
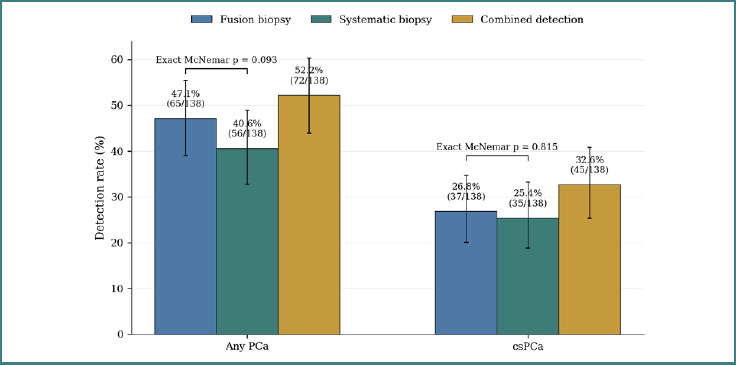
Detection rates for any prostate cancer and clinically significant prostate cancer. Bars represent observed proportions, and whiskers indicate 95% confidence intervals. Exact McNemar P values refer to paired comparisons between fusion biopsy and systematic biopsy. Combined detection indicates cancer detected by either biopsy method. csPCa, clinically significant prostate cancer; PCa, prostate cancer.

Clinically significant prostate cancer was detected by fusion biopsy in 37 patients (26.8%) and by systematic biopsy in 35 patients (25.4%). Combined biopsy detected clinically significant disease in 45 patients (32.6%). The paired difference between the two biopsy components was small and non-significant (exact McNemar *P* = 0.815), while the combined biopsy detected more clinically significant cancers than either component alone (Table 2, Figure 2).

**Table 2:** Overall and clinically significant prostate cancer detection

Outcome	Fusion biopsy	Systematic biopsy	Combined biopsy	Exact McNemar *P* value
Any prostate cancer	65/138 (47.1%)	56/138 (40.6%)	72/138 (52.2%)	0.093
Clinically significant prostate cancer	37/138 (26.8%)	35/138 (25.4%)	45/138 (32.6%)	0.815

The exact McNemar test compares paired detection rates between fusion biopsy and systematic biopsy. Combined biopsy indicates detection by either fusion biopsy or systematic biopsy. Clinically significant prostate cancer was defined as a Gleason score ≥3+4 / ISUP Grade Group ≥2.

### Concordance and discordance between biopsy methods

**Figure 3:**
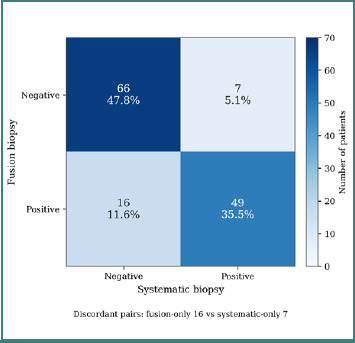
Paired concordance matrix for overall prostate cancer detection. Values represent the number and percentage of patients in each paired biopsy category. Discordant pairs consisted of 16 fusion-positive/ systematic-negative cases and 7 fusion-negative/systematic- positive cases.

At the patient level, both biopsy techniques were negative in 66 patients (47.8%) and positive in 49 patients (35.5%). Fusion biopsy was positive while systematic biopsy was negative in 16 patients (11.6%), whereas systematic biopsy was positive while fusion biopsy was negative in seven patients (5.1%) (Table 3, Figure 3).

**Table 3:** Concordance and discordance for overall prostate cancer detection

Fusion biopsy	Systematic biopsy	*n*	%
**Negative**	Negative	66	47.8
**Positive**	Positive	49	35.5
**Positive**	Negative	16	11.6
**Negative**	Positive	7	5.1
**Total**		138	100.0

Percentages were calculated using the full cohort as the denominator *(n* = 138). Fusion-positive/systematic-negative cases represent fusion-only detection, whereas fusion-negative/systematic-positive cases represent systematic-only detection.

Discordant results were therefore observed in 23 patients (16.7%). Fusion-only positive cases were more frequent than systematic-only positive cases, indicating a higher observed incremental contribution of MRI-directed sampling to overall cancer detection in this cohort.

### PI-RADS-stratified detection patterns

Detection rates increased in a stepwise manner with PI-RADS category. In PI-RADS 3 lesions, detection rates were low for both biopsy methods. In PI-RADS 4 lesions, fusion biopsy showed a numerically higher overall cancer detection rate than systematic biopsy, although clinically significant disease was detected at comparable frequencies by the two methods.

**Figure 4:**
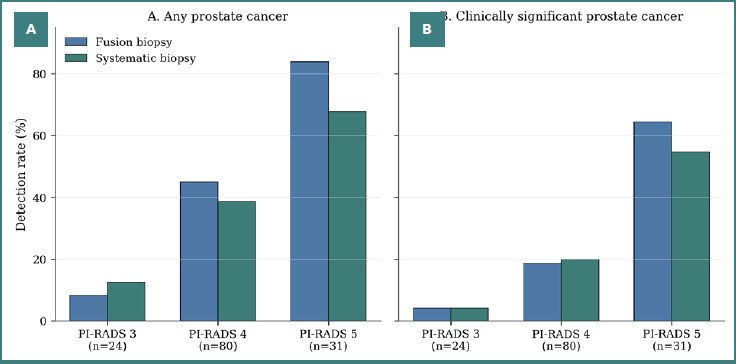
Detection rates according to PI-RADS category. **A**, overall prostate cancer detection; **B** shows clinically significant prostate cancer detection. Percentages were calculated within each PI-RADS category. csPCa, clinically significant prostate cancer; PI-RADS, Prostate Imaging Reporting and Data System.

The highest detection rates were observed in PI-RADS 5 lesions. In this subgroup, fusion biopsy detected overall prostate cancer in 26 of 31 patients (83.9%) and clinically significant disease in 20 of 31 patients (64.5%), compared with 21 of 31patients (67.7%) and 17 of 31 patients (54.8%), respectively, for systematic biopsy. The numerical difference between fusion and systematic biopsy was greatest in the PI-RADS 5 subgroup (Table 4, Figure 4).

**Table 4:** PI-RADS-stratified detection rates

PI-RADS category	*n*	Fusion any cancer	Systematic any cancer	Fusion csPCa	Systematic csPCa
PI-RADS 3	24	2/24 (8.3%)	3/24 (12.5%)	1/24 (4.2%)	1/24 (4.2%)
PI-RADS 4	80	36/80 (45.0%)	31/80 (38.8%)	15/80 (18.8%)	16/80 (20.0%)
PI-RADS 5	31	26/31 (83.9%)	21/31 (67.7%)	20/31 (64.5%)	17/31 (54.8%)

Percentages were calculated within each PI-RADS category. PI-RADS information was available for 135 patients. csPCa, clinically significant prostate cancer, defined as Gleason score ≥3+4 / ISUP Grade Group ≥2; PI-RADS, Prostate Imaging Reporting and Data System.

### Surgical pathology status and exploratory operatedpatient analysis

Surgical pathology was available in 45 patients who underwent radical prostatectomy. Among the remaining patients, 66 had no cancer on biopsy and were therefore classified as not applicable for surgical pathology, while 27 had cancer detected on biopsy but no surgical pathology available in the database. This latter category included patients with biopsy-detected cancer for whom no radical prostatectomy specimen was available in the study database (Table 5).

**Table 5:** Surgical pathology status

Surgical pathology status	*n*	Interpretation
Radical prostatectomy pathology available	45	Postoperative surgical pathology available
Not applicable: no cancer detected on biopsy	66	No biopsy-detected cancer; radical prostatectomy pathology not expected
Cancer detected on biopsy, surgical pathology unavailable	27	Biopsy-detected cancer, but no radical prostatectomy pathology available in the study database

Surgical pathology status was classified to distinguish unavailable postoperative pathology from the absence of disease. Surgical pathology analyses were restricted to patients with available radical prostatectomy specimens. cancer, defined as Gleason score ≥3+4 / ISUP Grade Group ≥2; PI-RADS, Prostate Imaging Reporting and Data System.

**Figure 5:**
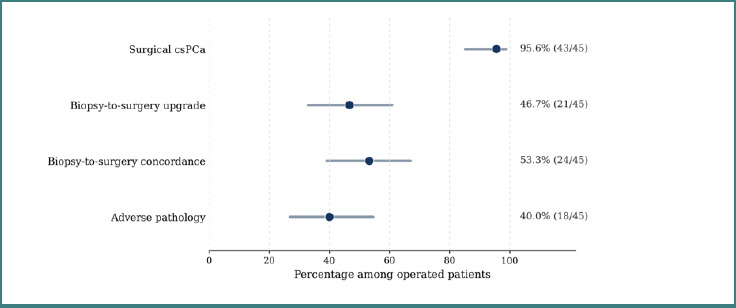
Key findings from the exploratory surgical pathology subgroup. Points represent observed proportions, and horizontal lines represent 95% confidence intervals. Percentages were calculated among patients with available radical prostatectomy pathology. Adverse pathology was defined according to the available postoperative pathology variable in the study database. csPCa, clinically significant prostate cancer.

Within the operated subgroup, clinically significant prostate cancer was confirmed on surgical pathology in 43 of 45 patients (95.6%). Biopsy-to-surgery upgrading occurred in 21 patients (46.7%), whereas concordance between the highest biopsy grade and final surgical grade was observed in 24 patients (53.3%). Adverse pathological features were identified in 18 patients (40.0%) (Table 6, Figure 5).

**Table 6:** Exploratory surgical pathology findings among patients who underwent radical prostatectomy.

Variable	Value
Patients with available radical prostatectomy pathology	45
Clinically significant prostate cancer on surgical pathology	43/45 (95.6%)
Biopsy-to-surgery upgrading	21/45 (46.7%)
Biopsy-to-surgery concordance	24/45 (53.3%)
Adverse pathology	18/45 (40.0%)
pN1 disease	3/44 (6.8%)
Lymphovascular invasion	7/45 (15.6%)
Perineural invasion	43/45 (95.6%)

Percentages were calculated among patients with available radical prostatectomy pathology unless otherwise specified. pN1 disease was calculated among patients with available nodal status. Adverse pathology was defined according to the available postoperative pathology variable in the study database. csPCa, clinically significant prostate cancer, defined as Gleason score ≥3+4 / ISUP Grade Group ≥2.

### Complications

**Figure 6:**
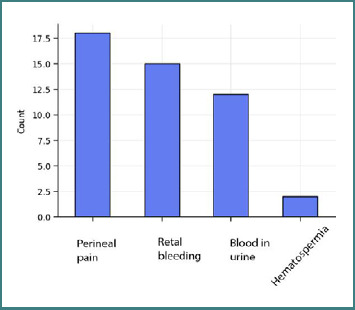
The distribution of the types of complications in the subgroup that had only mild complications

**Figure 7:**
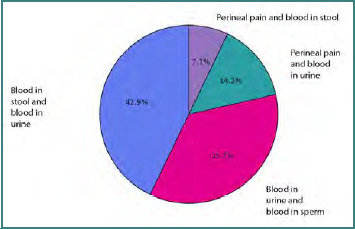
The distribution of the types of complications in the subgroup that had moderate complications

**Figure 8:**
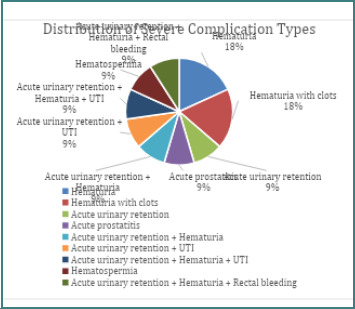
Chart depicting the distribution of complications in the subgroup that had severe complications

Complication severity was recorded for all patients. No complications were documented in 52 patients (37.7%), mild complications in 49 patients (35.5%), moderate complications in 25 patients (18.1%), and severe complications in 12 patients (8.7%). Because both biopsy components were obtained during the same diagnostic episode, complications were attributed to the biopsy episode as a whole rather than to either biopsy component separately (Table 7, Figures 6-8).

**Table 7:** Complication severity recorded after the biopsy episode

Severity	*n*	%
None	52	37.7
Mild	49	35.5
Moderate	25	18.1
Severe	12	8.7
Total	138	100.0

Percentages were calculated using the full cohort as the denominator *(n* = 138). Because fusion biopsy and systematic biopsy were performed during the same diagnostic episode, complications were attributed to the biopsy episode as a whole rather than to either biopsy component separately.

Complication data were analyzed descriptively, and no component-specific safety comparison between fusion biopsy and systematic biopsy was performed. The operational definitions of complication severity were based on the available retrospective source records.

## DISCUSSION

In this single-center paired cohort, MRI–ultrasound fusion biopsy yielded a numerically higher overall prostate cancer detection rate than systematic biopsy, with an absolute difference of 6.5 percentage points. The most informative observation, however, was the discordant-pair pattern: fusion biopsy identified 16 cancers in patients whose systematic biopsy was negative, whereas systematic biopsy identified seven cancers that were missed by fusion biopsy. This asymmetry suggests an observed incremental diagnostic contribution of fusion biopsy in patients with MRI-visible lesions [6,9]. Because the paired difference for overall cancer detection did not reach statistical significance, this finding should be interpreted as a numerical trend rather than definitive evidence of superiority.

The difference between the two methods was far less pronounced for clinically significant disease. Fusion biopsy and systematic biopsy detected clinically significant prostate cancer in 26.8% and 25.4% of patients, respectively, while the combined strategy detected such disease in 32.6%. These findings do not support a substitutional interpretation in which one biopsy technique fully replaces the other. Rather, they suggest that fusion biopsy improves lesion-directed sampling but does not fully replace the broader anatomical coverage provided by systematic biopsy [6,9,10].

The PI-RADS-stratified analysis reinforces this interpretation. Detection rates rose in parallel with MRI suspicion, and the clearest numerical advantage of fusion biopsy was seen in PIRADS 5 lesions. This result is biologically and clinically coherent because targeted biopsy is expected to perform best when the imaging target is highly suspicious and well-defined spatially. By contrast, in PI-RADS 3 lesions, both techniques detected relatively little cancer, underscoring the more uncertain biological significance of intermediate-risk MRI findings [3,5].

The present study aligns with the broader literature on MRI- guided diagnostics. Large contemporary studies have shown that targeted biopsy improves the detection of clinically significant prostate cancer, but also that systematic biopsy continues to contribute additional information in a subset of patients [4,6,9]. The current cohort echoes this pattern in a real-world single-center setting: fusion biopsy showed additional observed diagnostic yield, yet systematic biopsy remained diagnostically complementary.

The exploratory surgical pathology analysis adds an additional layer of clinical context. Among patients who underwent radical prostatectomy, the high prevalence of clinically significant disease, the frequency of biopsy-to-surgery upgrading, and the presence of adverse pathological features indicate that the surgically treated subgroup was enriched for clinically relevant tumors. These data are useful because they reflect the downstream clinical significance of the biopsy findings. At the same time, they must be interpreted cautiously because they derive from a selected subset rather than from the full cohort [11,12].

From a practical standpoint, the results are consistent with the value of a combined biopsy strategy in appropriately selected patients with suspicious mpMRI findings. Fusion biopsy improves sampling of MRI-visible lesions and increases overall cancer detection, particularly when MRI suspicion is high. Systematic biopsy complements targeted sampling by broadening glandular coverage and retaining the ability to identify MRI-invisible or non-targeted disease. The optimal balance between targeted-only and combined biopsy strategies will likely remain dependent on local expertise, imaging quality, patient characteristics, and lesion profile [3,6,9,13].

Several strengths of the study deserve mention. The within-patient paired design minimized confounding by patient-level heterogeneity; the analysis distinguished overall cancer detection from clinically significant disease; and surgical pathology findings were interpreted cautiously rather than overstated. The introduction of a dedicated surgical pathology status variable helped clarify that missing postoperative pathology did not imply negative disease.

Antibiotics remain central to the prevention and management of infectious complications after prostate biopsy, particularly in the transrectal setting, where rectal flora can seed the urinary tract, prostate, or bloodstream. Current evidence suggests that prophylaxis should be tailored to local resistance patterns and, when feasible, guided by rectal swab or urine culture results rather than relying on uniform empirical regimens. This is especially relevant given the rising prevalence of fluoroquinolone-resistant and multidrug-resistant organisms. In patients who develop post-biopsy fever, urinary tract infection, prostatitis, or suspected sepsis, prompt clinical assessment and early initiation of culture-directed antimicrobial therapy are essential, with escalation to broad-spectrum intravenous agents in severe cases while awaiting microbiological confirmation. At the same time, antibiotic stewardship remains important because unnecessarily prolonged or overly broad prophylaxis may contribute to resistance without improving outcomes. Recent literature also supports the transperineal route as a strategy associated with substantially lower infectious risk and, in many settings, reduced or even omitted antibiotic prophylaxis. Accordingly, the role of antibiotics should be viewed not in isolation, but as part of a broader complication-reduction strategy that includes biopsy route selection, local microbiological surveillance, and rapid recognition and treatment of post-procedural infection [14,15].

The study also has limitations. It is retrospective and single- center, and the sample size restricts the precision of subgroup analyses. Detailed procedural variables were not consistently available, including the number of targeted and systematic cores, fusion platform, sampling order, anesthesia, and antibiotic prophylaxis. Because these factors may influence both cancer detection and complication rates, this should be considered an important limitation of the retrospective design [16]. In addition, complication severity definitions were based on available source records and could not be further standardized retrospectively. From a data-governance perspective, retrospective clinical datasets remain valuable for describing local diagnostic pathways, but their use requires careful attention to data completeness, privacy, and regulatory standards, particularly in the Romanian context [17]. Finally, although biopsy histopathology was complete for the analytical cohort, surgical pathology remained limited to the operated subgroup; therefore, the study should be interpreted as a paired diagnostic-yield analysis rather than a full diagnostic-accuracy study using a universal reference standard.

## CONCLUSION

In this single-center paired analysis, MRI–ultrasound fusion biopsy showed a numerically higher overall prostate cancer detection rate than systematic biopsy, with the greatest benefit observed in PI-RADS 5 lesions. Systematic biopsy nevertheless retained complementary diagnostic value, including for clinically significant disease. Taken together, these findings are consistent with the value of a combined MRI-targeted and systematic biopsy approach in selected patients with suspicious mpMRI findings. In office-based transrectal prostate biopsy procedures, careful antibiotic stewardship appears to be a safe and appropriate approach to complication prevention and management. The postoperative pathological findings are clinically informative, but they should remain exploratory because they are limited to the operated subgroup.
